# Butyrate Derivatives Exhibited Anti-Inflammatory Effects and Enhanced Intestinal Barrier Integrity in Porcine Cell Culture Models

**DOI:** 10.3390/ani15091289

**Published:** 2025-04-30

**Authors:** Lauren Kovanda, Monika Hejna, Tina Du, Yanhong Liu

**Affiliations:** 1 Department of Animal Science, University of California, Davis, CA 95616, USA; llkovanda@gmail.com (L.K.); xdu@ucdavis.edu (T.D.); 2 Department of Biotechnology and Nutrigenomics, Institute of Genetics and Animal Biotechnology of the Polish Academy of Sciences, Postępu 36A, 05-552 Jastrzębiec, Poland; m.hejna@igbzpan.pl; 3 Department of Nutrition, University of California, Davis, CA 95616, USA

**Keywords:** butyrate, butyrate derivatives, IPEC-J2, porcine-alveolar macrophages, pro-inflammatory cytokines, transepithelial electrical resistance

## Abstract

Butyrate and its derivatives may influence inflammatory status and physiology in a variety of organisms and organ systems. Inflammatory conditions of the gastrointestinal tract, such as post-weaning diarrhea, negatively impact swine. Dietary intervention with butyrate-based compounds should be considered a strategy to improve disease resistance in pigs. We aimed to assess the properties of different forms of butyrate treatments using porcine cell culture experiments. This assessment may inform future in vivo feed experiments designed to determine its potential application of the dietary supplements for pigs. An intestinal porcine enterocyte cell line, IPEC-J2, was seeded at 5 × 10^3^ cells/mL in 96-well plates to confirm cell viability by MTT assay for each dose range used in the current experiments (0, 0.5, 1, 2, 4 mM butyric acid or tributyrin; 0, 1, 2, 4, 8 mM sodium butyrate or monobutyrin). For transepithelial electrical resistance (TEER) analysis, IPEC-J2 was seeded at 5 × 10^5^ cells/mL in 12-well transwell inserts and treated with 5 levels of each butyrate derivative after adherence (n = 5). TEER was measured at 24, 48, and 72 h post-treatment to quantify intestinal barrier integrity of IPEC-J2 monolayers. Butyric acid, sodium butyrate, and monobutyrin significantly increased (*p* < 0.05) TEER in IPEC-J2 at different time points compared with control. Further, porcine alveolar macrophages (PAMs) were harvested from donor weaned piglets (n = 6) via bronchoalveolar lavage and isolated for primary culture (6 × 10^5^ cells/well, 6-well plates). PAMs were treated with five levels of each butyrate derivative with or without lipopolysaccharide (LPS, 1 μg/mL) challenge. The concentrations of TNF-α and IL-1β in cell culture supernatants were measured by enzyme-linked immunosorbent assay (ELISA). Butyric acid and sodium butyrate treatments reduced the production of TNF-α in LPS-challenged PAMs (linear; *p* < 0.05). Different butyrate derivatives exerted anti-inflammatory properties and improved intestinal barrier integrity.

## Simple Summary

Butyrate is an organic acid compound present in the gastrointestinal environment which has been shown to positively influence gut health and reduce inflammation. Primarily, the presence of butyrate is understood to be a product of the beneficial microbes present in the intestinal environment. However, in the interest of directly administering this functional nutrient in diets, different derivatives such as glycerides and salts of the acid may be useful. In order to further characterize butyrate and its derivatives, this study aimed to evaluate several different butyrate-based compounds for effects on inflammation and barrier integrity in cells derived from pigs. The findings from these experiments confirm the efficacy of butyric acid as an anti-inflammatory agent and its impact on promoting the integrity of the intestinal barrier in vitro. In addition, other derivatives of butyrate, which are more manageable for handling and inclusion in pig diets, demonstrated comparable impacts on physiology in the cell culture models. This study provides evidence in support of the application of different butyrate derivatives as a dietary supplement to promote health in pigs.

## 1. Introduction

Butyric acid is a four-carbon short chain fatty acid produced by bacterial fermentation of fiber in the lower digestive tract of vertebrates by commensal microbial species diverse in phylogeny [1]. Short chain fatty acids, including butyrate, are metabolized by enterocytes of the intestine for energy supply, and these microbial products are vital for maintaining intestinal homeostasis [2]. Extensive research has determined the regulatory properties of butyrate in murine models and human cell lines, including anti-cancer effects [3,4,5] and regulation of cytokine production [6,7]. In addition, research using porcine-derived cells has identified the immunomodulatory effects of butyrate, such as the induction of host defense peptide production [8,9,10]. Previously, Yan and Ajuwon (2017) characterized the mitigatory effects on inflammation by sodium butyrate treatment in a porcine intestinal cell line, IPEC-J2. Sodium butyrate treatment protected against reduced expression tight junction proteins under lipopolysaccharide (LPS) challenge [11]. However, the literature lacks evidence supporting benefits conferred by glyceride forms of butyrate in vitro. Mono-, di- and triglycerides are liquid compounds composed of one, two, or three fatty acids, respectively, esterified to a glycerol backbone. Glycerides are known for a pleasant odor in contrast with the pungent odor of free fatty acids or their salt solutions [12]. These qualities afford fatty acid glycerides, such as monobutyrin and tributyrin, increased palatability when considered for dietary application in vivo. Dietary tributyrin has demonstrated positive impacts on piglet growth performance [13,14,15]. When included in the diet of pigs, other butyrate-based compounds have been shown to improve growth performance [16,17], reduce inflammatory status [18] and the incidence of diarrhea [19], and increase the expression of tight junction proteins [20]. These qualities should be considered when evaluating different forms of butyrate for feed additives in swine diets. Therefore, in the current study, we aimed to focus on the anti-inflammatory effects and impacts on intestinal barrier integrity by different butyrate derivatives in vitro.

The use of bacterial LPS challenge to polarize porcine alveolar macrophages (PAMs) into the pro-inflammatory M1 phenotype is an established in vitro model which can be utilized to observe changes in secreted cytokine levels [21,22,23]. IPEC-J2 is a commonly used in vitro model, exhibiting expression of tight junction proteins and microvilli similarly to the monolayer of enterocytes lining the intestine in vivo while remaining non-transformed [24,25]. The cell line differentiates into a monolayer that is polarized across the basal and apical aspects when cultured on transwell inserts containing membranes with 0.4 µm pores [26]. The transepithelial electrical resistance (TEER) across a monolayer can be measured to quantify epithelial integrity attributed to tight-junction-mediated conductance of ions through the paracellular route, where higher resistance is positively correlated with electric potential difference [27]. Equipped with cell culture models, the objectives of the present study were to determine the effects of butyric acid and three butyrate derivatives (sodium butyrate, monobutyrin, and tributyrin) on the intestinal barrier function and to explore their anti-inflammatory properties in vitro. We aimed to confirm and/or identify the influences of several forms of butyrate which may relate to their functionality as feed additives. Based on the existing literature regarding the regulatory function of butyrate and the benefits observed when it is included in swine diets, we hypothesized that butyrate derivatives would modulate inflammatory cytokine production and the intestinal barrier integrity of porcine-derived cell cultures. The current study evaluated the concentrations of two pro-inflammatory cytokines (TNF-α and IL-1β) secreted by PAMs under LPS-challenge conditions and measured the TEER in IPEC-J2 after treatment with several levels of butyric acid, sodium butyrate, monobutyrin, or tributyrin.

## 2. Materials and Methods

### 2.1. Butyrate Derivatives

All butyrate derivatives except monobutyrin were purchased from Sigma (St. Louis, MO, USA). The monobutyrin-based product was generously donated by Perstorp Waspik BV (Waspik, the Netherlands), which was a mixture of approximately 50% monobutyrin, 35% dibutyrin, 5% tributyrin, and 10% glycerol. To simplify the treatment name, monobutyrin was used to represent this product. 

Butyric acid is liquid in form and water-soluble (*ρ* = 1.135 g/mL) and was directly mixed into the cell culture medium at different tested concentrations. Sodium butyrate is also water-soluble and was used in solid form by dissolving directly into cell culture media to prepare a stock solution. Monobutyrin and tributyrin are in liquid form and not water-soluble. Therefore, monobutyrin was first dissolved into dimethyl sulfoxide (DMSO) and tributyrin was first dissolved into 100% ethanol prior to mixing into cell culture medium. The final concentration of DMSO in monobutyrin treatments did not exceed 0.5%, while the final concentration of ethanol in tributyrin treatments did not exceed 0.05%. 

All stock solutions were filtered with a sterile 0.22 µm filter to remove contaminants. Then, each solution was serially diluted further in cell culture media to prepare the treatments. The treatment doses were as follows: sodium butyrate and monobutyrin, 0.0, 1.0, 2.0, 4.0, 8.0 mM; butyric acid and tributyrin, 0.0, 0.5, 1.0, 2.0, 4.0 mM. These doses were selected for cell culture treatments based on preliminary cytotoxicity assays using IPEC-J2. The preparation procedures were utilized in all cell culture assays in the current research. 

### 2.2. Cell Culture Conditions

#### 2.2.1. IPEC-J2

IPEC-J2 were purchased from Leibniz Institute DSMZ-German Collection of Microorganisms and Cell Cultures GmbH (Braunschweig, Germany) and cultured with sterile Dulbecco Modified Eagle Medium (DMEM/F12) growth media supplemented with 1% antibiotics (100 IU penicillin/mL and 100 IU streptomycin/mL) and 5% heat-inactivated FBS. Live cells were stained by trypan blue dye and counted using a hemocytometer. For the TEER experiments, the final cell concentration was adjusted to 5 × 10^5^ cells/mL. Then, 0.5 mL of the IPEC-J2 cell suspension was seeded in each well of 12-well plates on 1.12 cm^2^ Corning polycarbonate transwell tissue-culture-treated inserts (0.4 µm pores; Corning Inc., Corning, NY, USA). The plates were then incubated at 37 °C in a humidified 5% CO_2_ incubator for adherence. The culture media were changed every other day and the cells were cultured for a total of 4 to 5 days until confluence was reached and the transepithelial electrical resistance (TEER) value was close to 1000 Ωcm^2^. All cells were then treated with 0.6 mL new media containing different doses of butyrate derivatives in the apical chamber of the transwell inserts. Treatment was performed in duplicate wells within 5 replicate 12-well plates. 

#### 2.2.2. Porcine Alveolar Macrophages

The protocol for this study was approved by the Institutional Animal Care and Use Committee (IACUC #20809) at the University of California, Davis. LPS (derived from *Escherichia coli* 0111:B4) was purchased from Sigma (St. Louis, MO, USA). PAMs were collected from six clinically healthy piglets at 7 weeks of age and BW of approximately 15 kg. The pigs were anesthetized by intramuscular injection of a 1 mL combination of telazol, ketamine, and xylazine (2:1:1) per 23.3 kg BW. The final mixture for anesthesia contained 100 mg telazol, 50 mg ketamine, and 50 mg xylazine in 1 mL (Zoetis Inc., Parsippany-Troy Hills, NJ, USA). After anesthesia, the pigs were euthanized by intracardiac injection with 78 mg sodium pentobarbital (Fatal-Plus; Med-Vet International, Mettawa, IL, USA) per 1 kg of BW.

PAMs were collected by bronchoalveolar lavage. First, the lungs with the intact trachea were removed immediately after euthanizing pigs without contaminating the interior of the trachea and the lungs with blood. Then, about 100 mL of sterile ice-cold phosphate-buffered saline without calcium or magnesium (PBS) was deposited into the lungs with a serological pipette through the trachea. After massaging the lungs for about 60 s, the lavage fluid was filtered through a double layer of sterile gauze into 50 mL conical centrifuge tubes. The cells were pelleted by centrifuging at 400 × *g* for 10 min at room temperature, then washed twice with ACK lysing buffer (Thermo Fisher, Cambridge, MA, USA) to lyse the red blood cells. The isolated macrophages were then resuspended in 5 mL Roswell Park Memorial Institute (RPMI-1640) growth medium with 1% antibiotics (100 IU penicillin/mL and 100 IU streptomycin/mL; Mediatech, Inc., Manassas, VA, USA) and 5% heat-inactivated fetal bovine serum (FBS; HyClone Laboratories, Inc., Logan, UT, USA). Live cells were stained by the trypan blue dye (Sigma-Aldrich Co., St. Louis, MO, USA) exclusion method and were counted using a hemocytometer (Fisher Scientific, Inc., Pittsburgh, PA, USA). The final cell concentration was adjusted to 10^6^ cells/mL. In this paper, the term, “porcine alveolar macrophages” is used because the majority of bronchoalveolar lavage fluid cells are macrophages [22].

Next, PAMs were cultured in 24-well cluster plates at a density of 6 × 10^5^ cells/well. The plates were incubated overnight at 37 °C in a humidified 5% CO_2_ incubator to allow macrophages to adhere to culture dishes. The nonadherent cells were washed away with pre-warmed RPMI-1640 medium. The adhered macrophages were treated in duplicate with fresh pre-warmed RPMI-1640 with FBS and antibiotics and treatments. After 24 h more of incubation, the supernatants in duplicates were collected, pooled, and stored at −80°C. This experiment tested all compounds with the same experimental design. The experimental design was a 2 (without or with 1 μg of LPS/mL) × 5 (different levels of tested compound) factorial arrangement in a randomized complete block design. Thus, there were a total of 10 treatments for each tested compound. The negative control was the treatment without butyrate derivatives or LPS, and the positive control was the treatment without butyrate derivatives but with LPS.

### 2.3. Cell Viability Assays

Cell viability assays were performed to determine the impacts of butyrate derivatives on the cell viability IPEC-J2 cells by a Vybrant MTT Cell Proliferation Assay Kit (Invitrogen Corporation, Carlsbad, CA, USA). MTT, a 3-(4,5-dimethylthiazol-2-yl)-2,5 diphenyltetrazolium bromide, measures the metabolic activity of cells via mitochondrial enzymes, which catalyze a color change reaction. Briefly, the cells prepared in each treatment were seeded at between 5 × 10^3^ and 10^4^ cells/mL in 96-well plates, and then incubated at 37 °C and 5% CO_2_ for 24 h. Then, all media were removed and replaced with 100 μL of fresh culture media and 10 μL of 12 mM MTT solution per well. After 4 h of incubation at 37 °C, all cell medium but 25 μL was removed from each well, and then 50 μL DMSO was added to reduce MTT to purple formazan crystals, creating a color change in proportion to the metabolic activity. After 10 min of incubation, the color change reaction was quantified by optical density (OD) measured at 540 nm with a reference wavelength of 630 nm (Synergy HTX Multi-Mode Microplate Reader, BioTek, Winooski, VT, USA). The delta OD values were calculated by the difference between the ODs of the two wavelengths. The negative control wells containing growth media without cells were used as a blank and subtracted from the delta absorbance obtained from each sample. The OD of positive control wells containing cells in growth media without treatment was used as a standard and set as 100% viability. The relative viability was calculated as follows:

(OD_treated cells_ − OD_blank_) × 100/(mean OD_positive control_) (1)

The percentage of live cells obtained for each treatment is a function of both viability and proliferation. A percentage of live cells below 50% indicates cytotoxic dosage. 

### 2.4. TEER Analysis

The TEER of IPEC-J2 cells was measured (Ωcm^2^) at 0 h (before treatment) and at 24, 48, and 72 h post-treatment using a Millicell ERS-2 voltohmmeter (MilliporeSigma, St. Louis, MO, USA). The experimental design was a 4 (different time points) × 5 (different levels of each compound) factorial arrangement in a randomized complete block design with 10 replicates in duplicate wells. Wells in duplicate containing transwell inserts and culture medium with no cells were used as blank measurements at each time point. Culture plates were allowed to reach room temperature before measurements in order to attain stable TEER readings. Electrodes were rinsed with 70% ethanol, 0.1 M NaCl solution, and finally pre-warmed medium between readings of wells containing different treatments. The resistance of each monolayer was calculated (Formula (2)) and was inversely proportional to the area of the membrane (Formula (3)), based on methodology by Srinivasan et al., (2015) [27]:

R_monolayer_ [Ω] = R_sample_ − R_blank_, (2)

R_monolayer_ [Ω] × monolayer area [cm^2^] = R_reported_ [Ωcm^2^] (3)

### 2.5. Proinflammatory Cytokines Analysis

Supernatants retrieved from PAMs were analyzed for the concentrations of TNF-α and IL-1β by commercial enzyme-linked immunosorbent assay (ELISA) kits (R&D Systems, Inc., Minneapolis, MN, USA). All procedures were performed according to the manufacturer’s instructions. Briefly, the standard, the control, and the samples were added to the wells with coated monoclonal antibody specific for each cytokine. After incubation for 2 h, the unbound substances were washed away, and an enzyme-linked polyclonal antibody specific for the cytokine was added to the wells to sandwich the cytokine immobilized during the first incubation. A further 2 h of incubation was followed by a wash to remove any unbound antibody–enzyme reagent, and then a substrate solution was added to the wells, and color was developed in proportion to the amount of the cytokine bound in the initial step. The color development was stopped by adding the stop solution, and the intensity of the color was measured at 540 nm. The concentrations were calculated from a standard curve. All samples were analyzed in duplicate. The detection limits of the ELISA kit for TNF-α and IL-1β analyses were 3.7 and 1.76 pg/mL, respectively.

### 2.6. Statistical Analyses

All data from the current study were analyzed in Rstudio (Rstudio, Boston, MA, USA, version 2023.06.2+561). Outliers were identified and removed based on the interquartile range method. All data were assessed for normality using the Shapiro–Wilk test or quantile–quantile plots. Homogeneity of variance was determined using Levene’s test. All data were analyzed by ANOVA. The least-squares means procedure was employed using the “emmeans” package in R (version 4.3.1) to calculate mean values and pairwise *t*-tests were performed to determine significant differences between the treatments. For TEER, linear dose responses were determined by orthogonal contrasts. LPS-challenged PAMs exhibited remarkably increased (*p* < 0.001) secretion of pro-inflammatory cytokines and were analyzed separately from PAMs treated without LPS. The statistical model for ELISA data included the dose as the independent variable and the donor pig as the random effect. For TEER, a pool of duplicate wells were the experimental unit and the statistical model included time, dose, and their interactions as fixed effects, with plate as a random effect. Within each timepoint, orthogonal contrasts were performed to identify linear dose responses. Probability values of <0.05 were considered significant.

## 3. Results

### 3.1. Cell Viability of IPEC-J2

The cell viability of IPEC-J2 was tested when treated with butyrate derivatives (Table 1.) Treatment with butyric acid resulted in no differences in cell viability. Lower doses (0.5, 1, or 2 mM) of tributyrin tended (*p* = 0.061) to increase the proliferation of IPEC-J2. Increased (*p* < 0.05) cell proliferation was also observed in IPEC-J2 treated with 2 or 4 mM of sodium butyrate, and 2 mM of monobutyrin.

### 3.2. TEER

**Figure 1:**
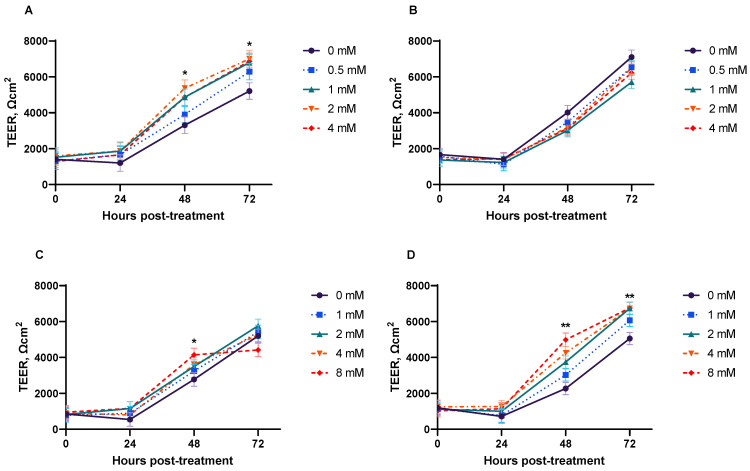
Butyrate derivatives influenced transepithelial electrical resistance (TEER) of IPEC-J2 cells. TEER is normalized by surface area of cell culture membranes (1.12 cm^2^) and therefore expressed in Ωcm^2^. An average blank TEER reading from each time point (wells with growth media and transwell inserts, but no cells) was subtracted from each value to calculate the reported TEER. For butyric acid (**A**), time: *p* < 0.001; dose: *p* < 0.01; interaction: *p* = 0.727. For tributyrin (**B**), time: *p* < 0.001; dose: *p* = 0.406; interaction: *p* = 0.936. For sodium butyrate (**C**), time: *p* < 0.001; dose: *p* = 0.523; interaction: *p* = 0.409. For monobutyrin (**D**), time: *p* < 0.001; dose: *p* < 0.001; interaction: *p* < 0.05. Orthogonal contrasts were performed to determine linear dose responses at each time point. * Dose response was linearly increased (*p* < 0.05) at that time point. ** Dose response was linearly increased (*p* < 0.001) at that time point. Each least square mean represents five replicate observations.

The TEER values in all IPEC-J2 increased (*p* < 0.001) over time (Figure 1). Significant dose effects were detected for butyric acid and monobutryin (*p* < 0.01 and *p* < 0.001, respectively), and a significant (*p* < 0.05) interaction between dose and time was observed in cells treated with monobutyrin. At 48 and 72 h post-treatment, butyric acid or monobutyrin treatments linearly increased (*p* < 0.05) the TEER of IPEC-J2 at dose responses (Figure 1A,D). At 48 h post-treatment, the highest dose of sodium butyrate linearly enhanced (*p* < 0.05) TEER in IPEC-J2 (Figure 1C). However, the tributyrin treatment had no effect on TEER in IPEC-J2 at the time points tested.

### 3.3. Pro-Inflammatory Cytokines from Porcine Alveolar Macrophages

**Figure 2:**
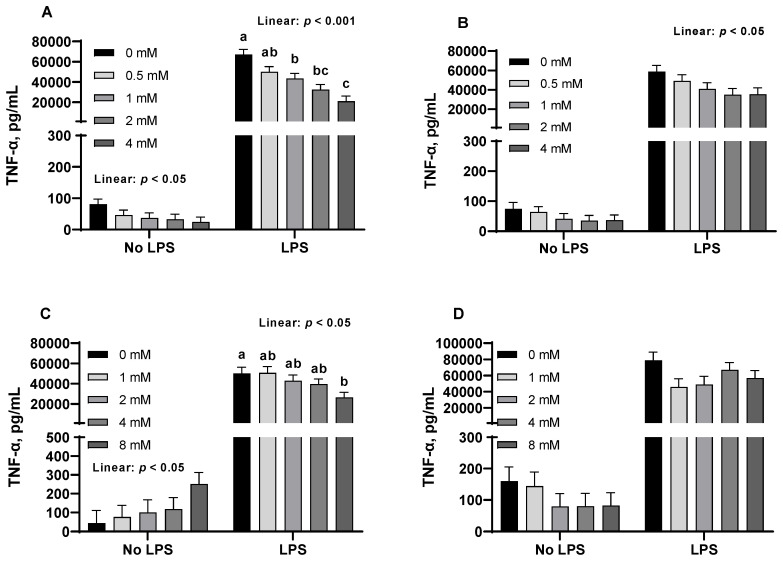
Butyrate derivatives influenced the production of tumor necrosis factor-alpha (TNF-α) from porcine alveolar macrophages (PAMs) in the presence of 1 μg/mL lipopolysaccharide (LPS), but not in the absence of LPS. The cells were incubated with various concentrations of each compound in the absence or presence of LPS (1 μg/mL) for 24 h. LPS significantly (*p* < 0.001) increased TNF-α secretion from PAMs. For treatment effects in the absence or presence of LPS, butyric acid (**A**), sham group: *p* = 0.158, LPS group: *p* < 0.001; tributyrin (**B**), sham group: *p* = 0.493, LPS group: *p* = 0.08; sodium butyrate (**C**), sham group: *p* = 0.210, LPS: *p* < 0.05; monobutyrin (**D**), sham group: *p* = 0.521, LPS group: *p* = 0.161. Letters (a–c) indicate significant (*p* < 0.05) differences among treatments in the presence of LPS. Orthogonal contrasts were performed to determine the linear dose responses of each butyrate derivative in the presence of LPS. Each least square mean represents five or six replicate observations.

**Figure 3:**
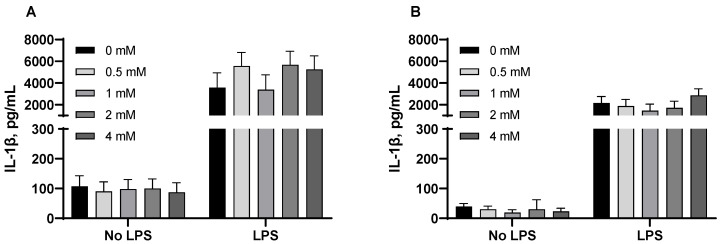

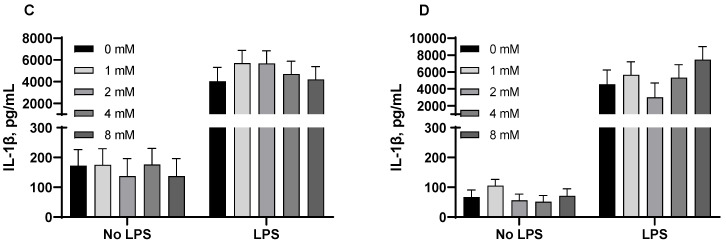
Butyrate derivatives did not influence the production of interleukin 1-beta (IL-1β) from porcine alveolar macrophages (PAMs) in the absence or presence of lipopolysaccharide (LPS). The cells were incubated with various concentrations of compound in the absence or presence of LPS (1 μg/mL) for 24 h. For all compounds, LPS significantly (*p* < 0.001) increased IL-1β secretion. For treatment effects in the absence or presence of LPS, butyric acid (**A**), sham group: *p* = 0.993, LPS group: *p* = 0.586; tributyrin (**B**), sham group: *p* = 0.898, LPS group: *p* = 0.549; sodium butyrate (**C**), sham group: *p* = 0.970, LPS group: *p* = 0.783; monobutyrin (**D**), sham group: *p* = 0.439, LPS group: *p* = 0.419. Each least square mean represents five or six replicate observations.

LPS challenge had significant impacts on TNF-α and IL-1β production from PAMs when comparing the non-challenged group with the challenged group (Figure 2 and Figure 3). Therefore, the dose effects were re-analyzed by ANOVA in the absence and presence of LPS, separately. In the absence of LPS challenge, no dose effects on secretion of pro-inflammatory cytokines were observed, except for a dose response (*p* < 0.05) to butyric acid and sodium butyrate in TNF-α production. In the presence of LPS challenge, butyric acid (1, 2, and 4 mM) and sodium butyrate (8 mM) significantly reduced (*p* < 0.05) TNF-α secretion from PAMs. In addition, orthogonal contrasts determined butyric acid, tributyrin, and sodium butyrate elicited a linear decrease (*p* < 0.05) in the production of TNF-α from PAMs challenged by LPS. However, the secretion of IL-1β by PAMs was not affected by butyrate derivatives in the absence or presence of LPS challenge (Figure 3).

## 4. Discussion

Several butyrate-based compounds were tested for their effects on barrier integrity and anti-inflammatory capacity in vitro. Determining whether butyrate delivered in salt or glyceride forms exerted these effects was of particular interest due to the reduced odor presented by these compounds compared with the acid form. Therefore, these derivatives of butyric acid may be more practically applied as potential feed additives for swine. Based on the results from the current study, butyric acid, sodium butyrate, and monobutyrin increased TEER in IPEC-J2, whereas all compounds besides monobutyrin elicited a linear dose response in TNF-α secretion by LPS-challenged PAMs. These results support in vivo outcomes, including reduced inflammatory status and the incidence of diarrhea in pigs fed butyrate-based supplements [18,19,28].

The barrier function of the intestinal epithelium is vital to maintaining host health. The intestinal epithelial lining is composed of a single layer of cells that prevents the bypass of microbial organisms and antigens. The cell population of the small intestine is diverse, including enterocytes, enteroendocrine cells, and immune cells [29,30]. Enterocytes make up the majority of the epithelium and perform various immune and regulatory functions, including the expression of Toll-like receptors (TLRs) and G-protein-coupled receptors (GPRs) [30,31,32]. However, their major function lies in innate immunity via maintaining a physical barrier, contingent on the proliferation and differentiation of enterocytes, which is modeled in vitro by IPEC-J2 monolayers. Previously, Zhao et al. (2021) reported no cytotoxic effects when IPEC-J2 was treated with up to 16 mM of sodium butyrate [33]. Similarly, sodium salts administered at physiological concentrations to several intestinal cell lines did not reduce viability, while butyric acid at the same concentrations was shown to be cytotoxic due to the induction of apoptotic pathways [34]. In the current study, we also observed that high doses (> 4 mM) of butyric acid and tributyrin reduced the viability of porcine epithelial cells. Thus, the dose responses of butyric acid and tributyrin on TEER were adjusted and tested up to 4 mM. Although tributyrin increased cell viability according to MTT results, surprisingly, TEER was not influenced by tributyrin treatment in the IPEC-J2 monolayers. The MTT assay reflects the mitochondrial activity of living cells. Butyrate may serve as a precursor to pyruvate destined for use in the tricarboxylic acid cycle [35]. However, the ability of tributyrin to form butyrate ions in cell culture systems is unknown and should be considered in future research. In the present study, sodium butyrate and monobutyrin at the lowest inclusion of 2 mM significantly increased the MTT values of IPEC-J2. Using HCT116 cells, butyrate has shown differential effects on cell proliferation according to the Warburg effect, which describes preferential glucose metabolism by cancerous cells and subsequent accumulation of butyrate [3]. Under glucose-deficient conditions, these cancer cells exhibited enhanced proliferation under low-dose (0.5-1 mM) butyrate treatment [3]. Among non-cancerous intestinal cells, butyrate has shown to upregulate the total activity of superoxide dismutase (SOD), an enzyme pivotal to the function of mitochondria [36,37]. According to Halliwell (2003), cell survival and division in culture systems undergo increased rates of reactive oxygen species production due to oxygen exposure [38]. The elevated percentages reported in the current study may be explained by this rationale, given the influence of butyrate on SOD activity [38]. Although the MTT assay has been widely used to analyze cell viability and proliferation, additional analyses, such as flow cytometric analysis and the bromodeoxyuridine cell proliferation assay, shall be considered in future research due to the limitations of the MTT assay. More in-depth analyses regarding the interactions of butyrate with the function of mitochondria, progression in cell cycle, and antioxidant activity should be explored.

It is known that microbial-produced butyrate in the large intestine serves as an energy source for enterocytes. Recently, the regulatory roles of butyrate in intestinal health have been identified. In the current study, sodium butyrate, butyric acid and monobutyrin significantly increased TEER in IPEC-J2 monolayers. These observations are in close agreement with previous reports, where sodium butyrate upregulated tight junction protein expression and TEER in human intestine epithelial cells [39,40] and porcine intestine epithelial cells [11,41]. Furthermore, butyrate treatment could stimulate the binding of transcription factor, specificity protein 1 (SP1), to the promoter region of *claudin-1* [42]. Thus, the enhancement in TEER by sodium butyrate may be claudin-dependent. TEER is a direct measurement of the resistance incurred by the tight junction barrier to passage of ions through the paracellular pathway of differentiated epithelial monolayers [27,43]. Therefore, the increased TEER observed with butyrate derivatives is likely related to the modulation of tight junctions. By reducing ion flux through the maintenance of tight junctions in the epithelium, butyrate derivatives may ameliorate the subsequent loss of water via the paracellular route during secretory diarrhea [44]. As such, the protection of barrier function during diarrhea by dietary supplementation with butyrate derivatives could be beneficial to organisms.

Moreover, the enhancement in TEER by butyric acid, sodium butyrate, and monobutyrin might be related to several other properties possessed by butyrate derivatives. Butyrate is a histone deacetylase (HDAC) inhibitor [45,46,47]. HDAC inhibition can alter the chromatin structure of DNA, leading to epigenetic modifications crucial for intestinal homeostasis [46,48,49]. Acetylation of histones and nucleosomal relaxation by butyrate compounds stimulated the transcription of genes associated with colonic epithelial cell maturation [50]. Another potential mechanism contributing to the enhancement in TEER is the activation of lipoxygenase by butyrate treatments. Previous research observed the upregulation of lipoxygenase-related genes concurrent with increased TEER in Caco-2 cells [39]. In the same study, the inhibition of lipoxygenase under butyrate-treated conditions significantly reduced TEER, suggesting the involvement of the lipoxygenase pathway in tight junction dynamics. The role of lipoxygenase in barrier function of IPEC-J2 monolayers, as well as other in vitro cultures of porcine intestine cells or tissues, should be further investigated. To date and to our knowledge, the production of brush border enzyme secretion by IPEC-J2 has not been well characterized [24]. Along these lines, limited impacts on TEER were observed in IPEC-J2 treated with tributyrin treatments compared with butyric acid, sodium butyrate, and monobutyrin. This observation may be attributed to the absence of pancreatic lipase or other esterases in vitro. It is possible that triglycerides were not cleaved, allowing for absorption by enterocytes, where energy may be directed to synthesis and functional localization of tight junction proteins [51]. However, the current study does not aim to draw implications regarding in vivo dietary butyrate supplementation, but instead to identify and confirm some basic properties among the tested derivatives. In addition, the triglyceride matrix may also impede the aforementioned non-nutrient properties of butyrate. Compared with butyrate, some structurally similar compounds such as beta-hydroxobutyrate exhibit reduced HDAC inhibition [52], suggesting the importance of chemical structure in the biological activities exerted by butyrate. 

Intestinal homeostasis is largely reliant on the regulation of inflammation. Uncontrolled inflammation during bacterial infections can be detrimental to host health. In the colon, microbially produced butyrate is considered an important mediator of homeostasis by modulating inflammation through incompletely understood mechanisms. In the current study, the secretion of pro-inflammatory cytokine, TNF-α, by porcine alveolar macrophages was reduced by butyric acid and sodium butyrate under LPS-challenge conditions. LPS binds the extracellular domain of TLR-4 dimers, inducing intracellular signaling to the phosphorylate inhibitor of Kappa B (IκB). This process releases the transcription factor Nuclear Factor (NF)-κB, allowing it to translocate to the nucleus and modulate the transcription of genes involved in the pro-inflammatory response, including TNF-α production [53,54]. Segain et al. (2000) demonstrated that butyrate inhibits the translocation of NF-κB to the nucleus under LPS challenge while maintaining IκB levels in monocytes [55]. In addition, butyrate has been shown to downregulate the expression of TLR-4 by enterocytes, congruent with the reduced production of pro-inflammatory cytokines by enterocytes [11]. HDAC inhibitors have been shown to downregulate the expression of TLRs and proinflammatory gene expression [56]. In macrophages, the NF-κB pathway may be inhibited by the reduced expression of TLR-4 when treated with butyrate, which would explain the reduced secretion of TNF-α in the current study. The production of TNF-α activates a variety of immune cell types and promotes further progression of the inflammatory response where, when uncontrolled, it has deleterious effects on intestinal barrier integrity [57] and mediates the pathogenesis of various diseases [58]. Interestingly, the production of another inflammatory mediator, IL-1β, was not impacted by butyrate derivatives under LPS challenge in the current study. Nod-like receptor protein 3 (NLRP3) inflammasome activation drives IL-1β production, which is mediated by internalized LPS, or an alternative pathway dependent on the activities of serine/threonine-protein kinase 1, FAS-associated death domain, and caspase-8 [59]. It is hypothesized that the integration of LPS in cytosol is a process independent of LPS activation of transmembrane receptors [60], and perhaps the LPS stimulation in the current cell culture experiment occurred without the internalization of LPS. On the other hand, the alternative activation of the NLRP3 inflammasome is inhibited by phenols present in cell culture media used in the current study, which may have contributed to the prevention of IL-1β production [61]. Additional cytokines produced downstream of LPS recognition, such as IL-6, are worth investigating in future research to better characterize how adaptive immunity may be influenced beyond the initial impacts on inflammatory markers. Other interactions between butyrate and intestinal physiology include its agonism of G-protein-coupled receptors, GPR41 and GPR43, which are expressed in immune cells and enterocytes and drive various downstream outcomes [62]. For instance, butyrate treatment in Chinese hamster ovary cells (CHOs) expressing GPR41 using a selectable marker system exhibited increased acetylation compared with GPR41-CHOs, suggesting GPR41-mediated HDAC inhibition [63]. In order to better understand and manipulate the complex relationship between intestinal function and butyrate, future research should aim to further characterize the pathways involved in augmenting TEER or reducing the production of TNF-α in porcine cell cultures.

## 5. Conclusions

Butyrate is a well-established anti-inflammatory agent with regulatory roles in the intestinal environment. The results from the current study are limited to the cell level in the absence of enzymatic and digestive activities which occur in vivo. As such, tributyrin had limited effects under in vitro conditions. Nonetheless, we have shown that sodium salt and monoglyceride forms of butyrate present similar in vitro properties compared with the pure acid form. Therefore, these compounds have the potential to be applied as feed additives for pigs. Weaned piglets benefit from dietary butyrate under inflammatory conditions in terms of improved growth performance and reduced incidence of diarrhea. These observations may be attributed to the modulation of enterocyte proliferation and tight junctions, as well as reduced production of TNF-α. Further investigation is required to confirm whether these modes of action are involved in disease resistance and improved intestinal health in vivo. Finally, we would like to recognize this work as a component of Dr. Lauren Kovanda’s PhD dissertation [64].

## Figures and Tables

**Table 1:** Cell viability (%) of IPEC-J2 treated with butyrate derivatives.

Dose (mM)	0	0.5	1	2	4	SEM	*p*-Value
Butyric acid	100.0	102.6	95.7	111.2	92.2	17.42	0.960
Tributyrin	100.0	142.2	125.6	146.1	91.7	17.24	0.061
Dose (mM)	0	2	4	8	16		
Sodium butyrate	100.0 ^b^	131.1 ^a^	129.1 ^a^	115.1 ^ab^	107.8 ^ab^	7.31	0.015
Monobutyrin	100.0 ^b^	129.2 ^a^	104.5 ^ab^	111.2 ^ab^	100.6 ^ab^	6.87	0.031

Superscript letters (^a, b^) indicate significant (*p* < 0.05) differences among treatments.

## Data Availability

The original contributions presented in this study are included in this article. Further inquiries can be directed to the corresponding author.

## Abbreviations

The following abbreviations are used in this manuscript:

ANOVA	Analysis of variance
CHOs	Chinese hamster ovary cells
DMEM	Dulbecco modified eagle medium
DMSO	Dimethyl sulfoxide
DNA	Deoxyribonucleic acid
ELISA	Enzyme-linked immunosorbent assay
FBS	Fetal bovine serum
GPR	G protein-coupled receptor
HDAC	Histone deacetylase
IL-1β	Interleukin 1-beta
IPEC-J2	Intestinal porcine enterocyte cell line
IκB	Inhibitor of Kappa B
LPS	Lipopolysaccharide
MTT	3-(4,5-dimethylthiazol-2-yl)-2,5-diphenyltetrazolium bromide
NF-κB	Nuclear factor kappa-light-chain-enhancer of activated B cells
NLRP3	Nod-like receptor protein 3
OD	Optical density
PAMs	Porcine alveolar macrophages
PBS	Phosphate buffered saline
RPMI	Roswell Park Memorial Institute
SOD	Superoxide dismutase
SP1	Specificity protein 1
TEER	Transepithelial electrical resistance
TLR	Toll-like receptor
TNF-α	Tumor necrosis factor-alpha
